# Feasibility of a craniometry in a comminuted zygomaticomaxillary complex fracture

**DOI:** 10.1097/MD.0000000000015839

**Published:** 2019-06-14

**Authors:** Shao-Yun Hsu, Pin-Keng Shih

**Affiliations:** a Department of Plastic and Reconstructive Surgery, China Medical University Hospital; b China Medical University, Taichung; c Department of Cosmetics and Health Care, Chung-Jen Junior College of Nursing, Health Sciences and Management, Chiayi City, Taiwan.

**Keywords:** C-arm imaging, craniometry, zygomaticomaxillary complex (ZMC) fracture

## Abstract

Few studies have reported on using craniometry for comminuted zygomaticomaxillary complex (ZMC) fracture management. We present our experiences with this procedure and a review of the related literature.

From September 2011 to October 2018, 43 patients with comminuted ZMC fracture receiving open reduction internal fixation under coronal incision were enrolled. Data on gender, age, operation time, hospital stay, duration of follow-up, vertical/horizontal differences, and complications were collected. Between-group differences (C-arm imaging vs craniometry) were evaluated using nonparametric Mann–Whitney test for continuous data and chi-square test for categorical data.

No significant difference were found between the groups regarding gender, age, hospital duration, follow-up duration, and postoperative complications, except for operation time. The averaged operation time was significantly shorter in the C-arm imaging group (4.217 h) than in the craniometry group (6.193 h). The C-arm imaging group had two cases with horizontal differences >3 mm and one case with vertical differences >3 mm. The craniometry group had four cases with horizontal differences >3 mm and four cases with vertical differences >3 mm. There were no significant differences between the two groups in horizontal differences and vertical differences.

Craniometry may achieve the same outcomes as C-arm imaging in comminuted ZMC fracture management; however, the former requires more time than the latter.

## 1 Introduction

Managing the treatment of a comminuted zygomaticomaxillary complex (ZMC) fracture is challenging for all surgeons. The inadequate reduction of ZMC fracture leads to asymmetrical facial contouring, diplopia, enophthalmos, facial numbness, and limited mouth opening. Traditial treatment approaches include subciliary or transconjunctival with a buccogingival incision, which will be adequate to reduce complications associated with a simple ZMC fracture. For complicated cases, accessory instruments such as intraoperative C-arm imaging,^[1]^ endoscopy,^[2]^ and computed tomography (CT)^[3]^ are options to assist with the proper alignment of the fracture site.

C-arm imaging is a popular means to reduce ZMC fracture complications. The advantages included immediate intraoperative imaging, fewer incisional wounds to achieve the same outcomes, and fewer operative and admission time, as well as lower learning curve for surgeons. Previous investigations have demonstrated good results in the reduction of zygomatic arch fracture^[4,5]^ and simple ZMC fracture.^[1]^ For comminuted ZMC fracture, the roles of the C-arm imaging are not as well characterized.

Craniometry is another option for symmetry reduction in ZMC fracture. No exposure to radiation and an easy operation are the main advantages to this procedure, but the precision measurements were easily affected by soft tissue swelling. Fewer studies regarding craniometry in comminuted ZMC fracture have been reported. In the present report, we have analyzed the outcomes in postoperative imaging of the craniometry and compared them with C-arm imaging. In addition, we have also evaluated which method is the best choice for the treatment of comminuted ZMC fractures.

## 2 Material and methods

This retrospective review was conducted at a single medical center between September 2011 and October 2018. The patients included in the analysis had a comminuted ZMC fracture with open reduction internal fixation (ORIF) under coronal incision as well as C-arm or craniometry performed during operation. The indication for patients with ORIF under coronal incision included simultaneous comminuted orbit rim and a lateral buttress of the maxilla and zygomatic arch. A total of 43 patients (C-arm imaging, n = 18; craniometry, n = 25) were included in the analysis. All the patients signed the informed consent.

### 2.1 Surgical technique

**Figure 1:**
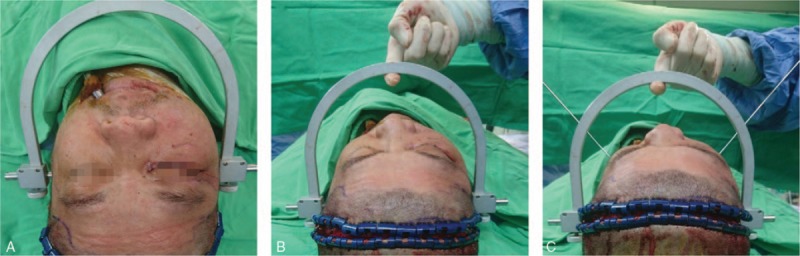
Craniometry. (A) A craniometry was set over bilateral ear canal. (B) The craniometry was kept straight by hand holding. (C) Two Kirschner pins were inserted from different position on the craniometry. After firm contact with the bone, the Kirschner pin was marked. The different lengths between two Kirschner pin marking points were measured.

A long incision line behind the hairline (approximately 4 cm) was made. The subgaleal plane was dissected using a scalpel after incision through the skin, subcutaneous tissue, and galea. Approximately 4 cm above the orbital rim, the incision (between the bilateral superior temporal lines) was made deep to the periosteum. In the temporal area, the superficial layer of the deep temporal fascia was dissected, and the superficial temporal fat pad was found. Simultaneously, an anterior and inferior dissection was made until the identification of the zygomatic arch was possible. Craniometry (Fig. 1) or the portable C-arm has used bilateral check symmetry after reduction. After facial fracture was attenuated, the detachment of the periosteum was repaired using Vicryl 3-0. Two Jackson–Pratt tubes were inserted subcutaneously. The scalp was repaired using skin staple. One month after discharge, a follow-up CT was performed.

### 2.2 Imaging survey

The horizontal and vertical measurements were referred from the study of Taehee Jo et al.^[6]^ Briefly, the line connecting the bilateral posterior margins of the fossa temporalis was defined as the horizontal baseline in the 3D reconstructed imaging. The horizontal distance was defined as between the most distant point on the anterior margin and the horizontal line. The horizontal difference was defined as the differences between the horizontal distance in the injured side and non-injured side.

The line connecting the bilateral supraorbital rim margins was defined as the baseline. The vertical distance was the length between the lowest point of the infraorbital and the baseline. The vertical difference was defined as the differences between the vertical distance in the injured side and non-injured side.

As reported previously,^[1]^ differences >3 mm between injured and uninjured side were considered clinically relevant. Two different plastic surgeons were enrolled for this measurement. If different findings were obtained from two plastic surgeons, another one was added.

### 2.3 Data collection

Data on age, gender, operation time, hospital days, follow-up duration, and postoperative complications of each patient were collected for statistical analysis.

### 2.4 Statistical analysis

The parameters were not normally distributed. Because the sample sizes between the two groups were different, the nonparametric Mann–Whitney test was used to determine the magnitudes of between-group differences. Chi-square test was used for determining differences between the two groups in gender, postoperative complications, and outcomes. Values of *P* < .05 were considered statistically significant. GraphPad for Windows version 5.01 was used for all statistical analysis.

## 3 RESULTS

### 3.1 The craniometry group

The study group consisted of 20 male and 5 female patients with a mean age of 39.83 years (range: 20–59 years). Operation time averaged to 6.193 h (range: 5–8 h), the hospital duration averaged to 11.17 days (range: 7–19 days), and the mean follow-up period was 10 months (range: 2–19 months).

There were 4 cases with horizontal differences >3 mm and 21 cases with differences <3 mm. There were 4 cases with vertical differences >3 mm and 21 cases with differences <3 mm.

One patient experienced a frontal branch injury, three cases with forehead numbness, three cases with widening incisional scar, and two patients had alopecia. The patient with frontal branch injury and forehead numbness received acupuncture therapy two times a week, and all symptoms improved half a year later. No treatments for widening incisional scar and alopecia were required.

### 3.2 The C-arm imaging group

The study group consisted of 16 male and 2 female patients with a mean age of 44.83 years (range: 20–70 years). Operation time averaged to 4.217 h (range: 3–5.3 h), the hospital duration averaged to 16.67 days (range: 6–26 days), and the mean follow-up period was 10.5 months (range: 3–20 months).

There were 2 cases with horizontal differences >3 mm and 16 cases with differences <3 mm. There was 1 case with vertical differences >3 mm and 17 cases with differences <3 mm.

To patients who experienced frontal branch injury, there were three cases with forehead numbness, two cases with widening incisional scar, and two patients with alopecia. The patient with frontal branch injury and forehead numbness received acupuncture therapy two times a week, and all symptoms improved half a year later. No treatments for widen incisional scar and alopecia were required by the patients.

### 3.3 Comparison of the two groups

There were no significant differences in age, gender, follow-up duration, hospital stay, postoperative complications, horizontal differences, and vertical differences between the two groups. There was a significant difference in operation time (4.217 vs 6.193 h, *P* < .05; Tables 1 and 2).

**Table 1:**
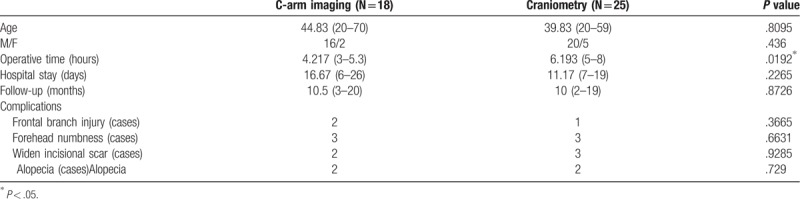
Characteristics of patients.

**Table 2:**
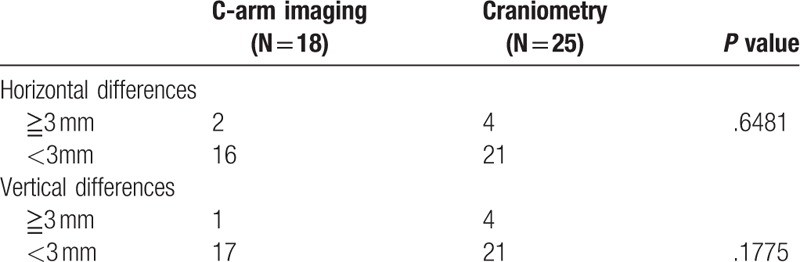
Outcomes of patients with C-arm imaging and craniometry.

## 4 Discussion

For comminuted ZMC fractures, it is often challenging to well maintain the projection and height of the zygoma well without adequate reduction of a tetrapod. The coronal incision is highly recommended for both comminuted ZMC and arch fractures because it affords a sufficient operative field. Even so, the bone chip loss in the fractured site sometimes made subsequent operation possible. Instantaneously intraoperative imaging such as CT or navigation guidance system may reduce the risks. However, the revision of the reduction of the zygoma was performed in 18% (95% CI 10.5–29.0%), and the revision of the orbital floor was performed in 9% (95% CI 3.6–17.2%).^[7]^

Intraoperative C-arm imaging appears to be a good option in the reduction of a zygomatic arch or simple ZMC fracture.^[8,9]^ The surgeons could make zygomatic arch fracture well reduction with immediate imaging no matter of simple or comminuted arch fracture.^[8]^ For a simple ZMC fracture, one-point fixation with immediate C-arm imaging could achieve the same effects as two-point fixations.^[8]^ The advantages of the above mentioned C-arm imaging technique are fewer incisional wounds, decreased operative and admission duration, fewer opportunities for secondary operation, and decreased cost of postoperative imaging. Although an initial learning curve is necessary, it made the inexperienced surgeons more confident in the management of ZMC fracture. However, the one-point fixation of ZMC fracture or simple reduction of zygomatic arch fracture without fixation may be made fractured site instability. To obtain a clear zygomatic view, neck hypertension increased the airway obstruction risks. Also, the radiation may be a burden to medical staff.

For comminuted ZMC fractures, although the coronal incision affords a clear and large operative field, the intraoperative C-arm imaging use not only made bilateral reduction symmetry but also decreased risks of the secondary operation. In this study, there were 16 cases (89%) with intraoperative C-arm imaging achieved bilateral horizontal symmetry (horizontal differences <3 mm), and 17 cases (94%) achieved bilateral vertical symmetry (vertical differences <3 mm). These data suggest that intraoperative C-arm imaging is a good option to achieve bilateral symmetry even in the comminuted ZMC fracture.

The craniometry is another option to achieving bilateral symmetry for reduction of ZMC fractures especially when non-portable C-arm imaging is unavailable. Compared with C-arm imaging, the craniometry is easy to perform and requires no exposure to radiation. However, the precision of measurement may be affected by soft tissue swelling. Therefore, the facial skin may be damaged by the sharp tip of Kirschner pin to get delicate measurements. Due to unavailable immediate imaging, repetitive multiple-points fixation followed by modifications with craniometry is necessary, which may take more operation time. Any unsymmetrical position of craniometry may lead to wrong interpretation. According to our experiences, the reduction suggested in 2 weeks after trauma could decrease the bias of measurement.

In this study, using craniometry, bilateral horizontal symmetry (horizontal differences <3 mm) was achieved in 21 (84%) cases and bilateral vertical symmetry (vertical differences <3 mm) in 21 (84%) cases. These data suggest that the craniometry was a good option to achieve bilateral symmetry even in the comminuted ZMC fracture.

There were no statistically significant differences in gender, hospital stay, follow-up duration, and complication rate between the C-arm imaging group and craniometry group except for the operation time. The averaged operation time (4.217 h) in the C-arm imaging group is significantly less than that (6.193 h) in the craniometry group under the C-arm imaging, the immediate imaging guide the operator to the well reduction of the fracture. On the contrary, the operator has to repeat the internal fixation and symmetrical measurement with craniometry for several times. It may explain why the craniometry group took more time than the C-arm imaging group in the reduction of the facial fracture.

According to the previous study, we considered the differences >3 mm between injured and uninjured side are clinically relevant.^[1]^ Although the case number in craniometry with larger vertical and horizontal differences is more than the C-arm imaging group, there were no statistically significant differences between the C-arm imaging and craniometry groups. This may imply the craniometry group could achieve the same outcome as the C-arm imaging group even the symmetrical measurement by craniometry may sometimes be affected by soft tissue swelling. There were two cases and one case with vertical and horizontal differences >3 mm in the C-arm imaging group, respectively. The inadequate neck hyperextension during operation left the zygomatic arch view some bias, which made some cases unsymmetrical in the following imaging.

One potential limitation of this investigation is the limited number of cases enrolled in both groups, and the procedures were performed at a single facility and may not be reflective of all institutions. A follow-up investigation including a larger number of participants is therefore warranted.

## 5 Conclusion

In conclusion, the craniometry could achieve the same bilateral symmetry as the C-arm imaging, but more operation time is required.

## Author contributions

**Conceptualization:** Shao-Yun Hsu, Pin-Keng Shih.

**Data curation:** Shao-Yun Hsu.

**Formal analysis:** Shao-Yun Hsu.

**Investigation:** Shao-Yun Hsu.

**Methodology:** Shao-Yun Hsu.

**Resources:** Pin-Keng Shih.

**Software:** Pin-Keng Shih.

**Supervision:** Pin-Keng Shih.

**Writing – original draft:** Shao-Yun Hsu.

**Writing – review & editing:** Pin-Keng Shih.
